# Supplementary material for: Quantum metrology with quantum-chaotic sensors
Source: Nat Commun. 2018 Apr 10;9:1351. doi: 10.1038/s41467-018-03623-z (PMC5893654; doi:10.1038/s41467-018-03623-z)
Supplement: Supplementary file 1 — Supplementary Information [file 41467_2018_3623_MOESM1_ESM.pdf]

# Quantum metrology with quantum-chaotic sensors

Fiderer et al.

**Supplementary Information**

**Supplementary Note 1. Realization of the kicked top**

The kicked top (KT) has been realized experimentally by Chaudhury et al. [1] using the atomic spin of a $^{133}$Cs atom in the $f = 3$ hyperfine ground state. Linear precession of the spin was implemented through magnetic pulses, and the torsion through an off-resonant laser field that exploited a spin-dependent rank-2 (ac Stark) light shift.

An implementation of the KT using microwave superradiance was proposed by Haake [2]: The top is represented by the collective pseudo-spin of $N$ two-level atoms coupled with the same coupling constant to a single mode of an electromagnetic field in a cavity, with a controlled detuning between mode and atomic frequencies. Large detuning compared to the Rabi frequency $\Omega = g\sqrt{N}$ with coupling strength $g$ allows one to adiabatically eliminate the cavity mode and leads to an effective interaction of the type $J_z^2$ [3] (replacing our $J_y^2$), while superradiant damping as described by the master equation (13) in the main text for the reduced density operator of the atoms can still prevail [2]. Finally, a linear rotation about the $x$-axis can be achieved through resonant microwave pulses, replacing the linear precession about the $z$-axis of the KT. The parameter $\alpha$ is now proportional to the Rabi frequency of the microwave pulse.

**Supplementary Note 2. Spin-exchange-relaxation-free Cs-vapor magnetometer**

Adapting standard notation in atomic physics, atomic spin operators will be denoted in the following by $\mathbf{F} = (F_x, F_y, F_z)$ with spin size $f$, $F_z|fm\rangle = m|fm\rangle$, and total electronic angular momentum $\mathbf{J} = \mathbf{L} + \mathbf{S}$ with quantum number $j$, composed of orbital angular momentum $\mathbf{L}$ and electron spin $\mathbf{S}$. We model a room-temperature spin-exchange-relaxation-free (SERF) Cs-vapor magnetometer similar to the experiments with Rb-vapor of Balabas et al. [4]. The Cs spin sensitive to the magnetic field $B$ is composed of a nuclear spin $K = 7/2$ and one valence electron with an electronic spin $s = 1/2$ which splits the ground state $6^2\mathrm{S}_{1/2}$ into two energy levels with total spin $f_1 = 3$ and $f_2 = 4$. This results in an effective Hilbert space of dimension $2(2K + 1) = 16$ for our model of a kicked SERF magnetometer.

The dominant damping mechanisms are related to collisions of cesium atoms with each

other and with the walls of the vapor cell.

In the SERF regime the spin-exchange rate is much greater than the rate of Larmor precession, typically realized by very small magnetic fields, a high alkali-atom density ($10^{13}$ atoms per $\mathrm{cm}^3$), high buffer-gas pressure, and heating of the vapor cell. Then, spin-exchange relaxation is so strong, that the population of hyperfine ground levels ($f_i = 3, 4$) is well described by a spin-temperature distribution. Here, we model a SERF magnetmeter with a lower alkali-atom density of $2 \times 10^{10}$ atoms per $\mathrm{cm}^3$ without buffer gas at room temperature $T = 294.13\,\mathrm{K}$. Modern alkene-based vapor-cell coatings support up to $10^6$ collisions before atoms become depolarized [4]. For a spherical vapor-cell with a 1.5 cm radius it follows that collisions with walls limit the lifetime of spin polarization to $T_{\mathrm{wall}} \simeq 92\,\mathrm{s}$. Since the effect of collisions among Cs atoms leads to stronger depolarization we neglect collisions with the walls in our model.

While the typical treatment proceeds by eliminating the nuclear-spin component we are interested in a dynamics that exploits the larger Hilbert space of the Cs spin. Therefore the evolution of the spin density matrix $\rho$ is described by a master equation that includes damping originating from collisions of Cs atoms [5] and an interaction with an off-resonant light field in the low-saturation limit [6] modeling the kicks:

$$\frac{d\rho}{dt} = R_{\mathrm{se}}\left[\varphi(1 + 4\langle \mathbf{S}\rangle \cdot \mathbf{S}) - \rho\right] + R_{\mathrm{sd}}\left[\varphi - \rho\right] + a_{\mathrm{hf}}\frac{[\mathbf{K}\cdot\mathbf{S}, \rho]}{i\hbar} + \frac{H_{\mathrm{A}}^{\mathrm{eff}}\rho - \rho H_{\mathrm{A}}^{\mathrm{eff}\dagger}}{i\hbar} + \gamma_{\mathrm{nat}}\sum_{q=-1}^{1}\left(\sum_{f,f_1} W_q^{ff_1}\rho_{f_1f_1}\left(W_q^{ff_1}\right)^\dagger + \sum_{f_1\neq f_2} W_q^{f_2f_2}\rho_{f_2f_1}\left(W_q^{f_1f_1}\right)^\dagger\right) \tag{1}$$

The first two summands describe spin-exchange relaxation and spin-destruction relaxation, respectively, where $R_{\mathrm{se}}$ denotes the spin-exchange rate and $R_{\mathrm{sd}}$ the spin-destruction rate, and $\varphi = \rho/4 + \mathbf{S}\cdot\rho\mathbf{S}$ is called the purely nuclear part of the density matrix, where the electron-spin operator $\mathbf{S}$ only acts on the electron-spin component with expectation value $\langle\mathbf{S}\rangle = \mathrm{tr}[\mathbf{S}\rho]$. The third summand is the hyperfine coupling of nuclear spin $\mathbf{K}$ and electronic spin $\mathbf{S}$ with hyperfine structure constant $a_{\mathrm{hf}}$, and the fourth summand drives the dynamic with an effective non-hermitian Hamiltonian on both ground-state hyperfine manifolds $H_{\mathrm{A}}^{\mathrm{eff}} = H_{\mathrm{A},f=3}^{\mathrm{eff}} + H_{\mathrm{A},f=4}^{\mathrm{eff}}$, with

$$H_{\mathrm{A},f}^{\mathrm{eff}} = \hbar\Omega_{\mathrm{Lar}}F_y + \sum_{f'}\frac{\hbar\Omega^2 C_{j'f'f}^{(2)}}{4(\Delta_{ff'} + i\gamma_{\mathrm{nat}}/2)}\left|\boldsymbol{\epsilon}_{\mathrm{L}}\cdot\mathbf{F}\right|^2 \tag{2}$$

that includes Larmor precession with frequency $\Omega_{\mathrm{Lar}} = g_f\mu_{\mathrm{B}}B/\hbar$ (with the Landé g-factor $g_f$

and the Bohr magneton $\mu_{\mathrm{B}}$) of the atomic spin in the external magnetic field $\mathbf{B} = B\hat{\mathbf{y}}$, and the rank-2 light-shift induced by a light pulse that is linearly polarized with unit polarization vector $\boldsymbol{\epsilon}_{\mathrm{L}}$ of the light field and off-resonant with detuning $\Delta_{ff'}$ from the D1-line transition with $f \to f'$. Further, we have the characteristic Rabi frequency $\Omega = \gamma_{\mathrm{nat}}\sqrt{I_{\mathrm{kick}}/(2I_{\mathrm{sat}})}$ of the D1 line, the natural line width $\gamma_{\mathrm{nat}}$, kick-laser intensity $I_{\mathrm{kick}}$, saturation intensity $I_{\mathrm{sat}}$, and the coefficient

$$C^{(2)}_{j'f'f} = (-1)^{3f-f'} \frac{\sqrt{30}(2f'+1)}{\sqrt{f(f+1)(2f+1)(2f-1)(2f+3)}} \begin{Bmatrix} f & 1 & f' \\ 1 & f & 2 \end{Bmatrix} \left| o^{j'f'}_{1/2f} \right|^2 , \tag{3}$$

where the curly braces denote the Wigner $6j$ symbol and

$$o^{j'f'}_{jf} = (-1)^{f'+1+j'+K} \sqrt{(2j'+1)(2f+1)} \begin{Bmatrix} f & K & j' \\ j & 1 & f \end{Bmatrix} , \tag{4}$$

where total angular momentum of ground and excited levels of the D1 line are $j = j' = 1/2$. Photon scattering is taken into account by the imaginary shift of $\Delta_{ff'}$ in the effective Hamiltonian and by the remaining parts of the master equation that correspond to optical pumping which leads to cycles of excitation to the $6\mathrm{P}_{1/2}$ manifold and spontaneous emission to the ground-electronic manifold $6\mathrm{S}_{1/2}$. When the laser is switched off the master equation solely involves the first four summands where $H^{\mathrm{eff}}_{\mathrm{A}}$ reduces to the Larmor precession term.

The jump operators are given as

$$W^{f_b f_a}_q = \sum_{f'=3}^{4} \frac{\Omega/2}{\Delta_{f_a f'} + i\gamma_{\mathrm{nat}}/2} \left(\mathbf{e}^*_q \cdot \mathbf{D}_{f_b f'}\right) \left(\boldsymbol{\epsilon}_{\mathrm{L}} \cdot \mathbf{D}^\dagger_{f_a f'}\right) , \tag{5}$$

with the spherical basis $\mathbf{e}_1 = -(\hat{\mathbf{x}} + i\hat{\mathbf{y}})/\sqrt{2}$, $\mathbf{e}_0 = \hat{\mathbf{z}}$, $\mathbf{e}_{-1} = (\hat{\mathbf{x}} - i\hat{\mathbf{y}})/\sqrt{2}$ in the Cartesian basis $\hat{\mathbf{x}}, \hat{\mathbf{y}}, \hat{\mathbf{z}}$, and the raising operator $\mathbf{D}^\dagger_{ff'} = \sum_{q,m,m'} \mathbf{e}^*_q o^{j'f'}_{jf} \left\langle f'm' | fm; 1q \right\rangle |f'm'\rangle\langle fm|$ with Clebsch-Gordan coefficients $\langle f'm'|fm; 1q\rangle$ and magnetic quantum numbers $m$, $m'$.

Excited state hyperfine levels are Doppler and pressure broadened, but we neglect pressure broadening which is much smaller than Doppler broadening due to the very low vapor pressure. Doppler broadening is taken into account by numerically averaging the righthand side of the master equation (Supplementary Equation 1) over the Maxwell-Boltzmann distribution of velocities of an alkali atom. This translates into an average over detunings $\Delta_{ff'}$. Since $\Delta \gg \Omega$ must hold within the description of this master equation, we limit averaging over detunings to a $3\sigma$ interval.

By numerically solving the non-linear trace-preserving master equation (Supplementary Equation 1) with the Euler method (analog to Ref. [7] we take hyperfine coupling into account by setting off-diagonal blocks of the density matrix in the coupled $|fm\rangle$-basis after each Euler step to zero, because they oscillate very quickly with $a_{\mathrm{hf}}$), we simulate dynamics similar to the dissipative kicked top described above with the difference that kicks are not assumed to be arbitrarily short, i.e. kicks and precession coexist during a light pulse. Kicks and corresponding dissipation are factored in by applying a superoperator to the state in each Euler step during a kick.

Spin-exchange and spin-destruction rates are estimated to $R_{\mathrm{se}} \simeq 12\,\mathrm{Hz}$ and $R_{\mathrm{sd}} \simeq 0.12\,\mathrm{Hz}$ from the known cross sections of Cs-Cs collisions, the mean relative thermal velocity of Cs atoms and their density.

For the concrete example of Fig. 7 we calculate with $2 \times 10^{10}$ Cs atoms per $1\,\mathrm{cm}^3$ vapor volume, and kick laser pulses linearly polarized in $x$-direction, $\boldsymbol{\epsilon}_{\mathrm{L}} = \hat{\mathbf{x}}$, with intensity $I_{\mathrm{kick}} = 0.1\,\mathrm{mW/cm}^2$ and detuning halfway between the two components of the D1 line, and $\Delta_{34} \simeq -584\,\mathrm{MHz}$. The period is $\tau = 1\,\mathrm{ms}$ where during the last $2\,\mu\mathrm{s}$ of each period the laser pulse is applied (effective kicking strength for the lower hyperfine level of the ground state is $k \simeq 6.5 \times 10^{-4}$). We choose a small magnetic field $B = 40\,\mathrm{fT}$ in $y$-direction so that we are well within the SERF regime, $R_{\mathrm{se}} \gg \Omega_{\mathrm{Lar}}$.

With a circular polarized pump beam in $z$-direction resonant with the D1 line the initial spin-state is polarized which in the presence of spin-relaxation leads to an effective thermal state

$$\rho = \frac{e^{\beta K_z} e^{\beta S_z}}{Z_K Z_S}, \tag{6}$$

with the partition sum $Z_j = \sum_{m=-j}^{j} e^{\beta m}$ and $\beta = \ln \frac{1+q}{1-q}$, with polarization $q = 0.95$. The readout is accomplished typically with the help of an off-resonant probe beam by measuring its polarization after it experienced a Faraday rotation when interacting with the atomic spin ensemble.

**Supplementary References**

[1] Chaudhury, S., Smith, A., Anderson, B., Ghose, S. & Jessen, P. S. Quantum signatures of chaos in a kicked top. *Nature* **461**, 768–771 (2009).

[2] Haake, F. Can the kicked top be realized? *Journal of Modern Optics* **47**, 2883–2890 (2000).

[3] Agarwal, G., Puri, R. & Singh, R. Atomic Schrödinger cat states. *Phys. Rev. A* **56**, 2249–2254 (1997).

[4] Balabas, M., Karaulanov, T., Ledbetter, M. & Budker, D. Polarized Alkali-Metal Vapor with Minute-Long Transverse Spin-Relaxation Time. *Phys. Rev. Lett.* **105**, 070801 (2010).

[5] Appelt, S. *et al.* Theory of spin-exchange optical pumping of $^3$He and $^{129}$Xe. *Phys. Rev. A* **58**, 1412–1439 (1998).

[6] Deutsch, I. H. & Jessen, P. S. Quantum control and measurement of atomic spins in polarization spectroscopy. *Optics Communications* **283**, 681–694 (2010).

[7] Savukov, I. & Romalis, M. Effects of spin-exchange collisions in a high-density alkali-metal vapor in low magnetic fields. *Phys. Rev. A* **71**, 023405 (2005).
